# Crystal Structures of the FAK Kinase in Complex with TAE226 and Related Bis-Anilino Pyrimidine Inhibitors Reveal a Helical DFG Conformation

**DOI:** 10.1371/journal.pone.0003800

**Published:** 2008-11-24

**Authors:** Daniel Lietha, Michael J. Eck

**Affiliations:** 1 Department of Biological Chemistry and Molecular Pharmacology, Harvard Medical School, Boston, Massachusetts, United States of America; 2 Department of Cancer Biology, Dana-Farber Cancer Institute, Boston, Massachusetts, United States of America; Ordway Research Institute, United States of America

## Abstract

Focal Adhesion Kinase (FAK) is a non-receptor tyrosine kinase required for cell migration, proliferation and survival. FAK overexpression has been documented in diverse human cancers and is associated with a poor clinical outcome. Recently, a novel bis-anilino pyrimidine inhibitor, TAE226, was reported to efficiently inhibit FAK signaling, arrest tumor growth and invasion and prolong the life of mice with glioma or ovarian tumor implants. Here we describe the crystal structures of the FAK kinase bound to TAE226 and three related bis-anilino pyrimidine compounds. TAE226 induces a conformation of the N-terminal portion of the kinase activation loop that is only observed in FAK, but is distinct from the conformation in both the active and inactive states of the kinase. This conformation appears to require a glycine immediately N-terminal to the “DFG motif”, which adopts a helical conformation stabilized by interactions with TAE226. The presence of a glycine residue in this position contributes to the specificity of TAE226 and related compounds for FAK. Our work highlights the fact that kinases can access conformational space that is not necessarily utilized for their native catalytic regulation, and that such conformations can explain and be exploited for inhibitor specificity.

## Introduction

Focal Adhesion Kinase (FAK) is a non-receptor tyrosine kinase that regulates signals involved in cell proliferation, migration and survival [1], [2]. Following cell adhesion, FAK is recruited to focal adhesions via its C-terminal focal adhesion targeting (FAT) domain [3] and activated by signals from growth factor and integrin receptors [2]. FAK activation is initiated by breaking an intramolecular autoinhibitory interaction between the N-terminal FERM (4.1, ezrin, radixin, moesin homology) and kinase domains [4]. This results in rapid autophosphorylation of Tyr397 in the linker between the FERM and kinase domains, recruitment of Src to pTyr397 and phosphorylation of the activation loop by Src. Src also phosphorylates tyrosines at the C-terminus of FAK, which contains docking sites for adaptor proteins like Grb2 and Cas. Hence, FAK exhibits dual functionality in focal adhesions as a signaling and a scaffolding molecule.

FAK is overexpressed in many tumors including those of the brain, ovary, colon, breast, prostate, liver and thyroid [5]–[10]. Furthermore, FAK overexpression is highly correlated with an invasive phenotype in these tumors. Inhibition of FAK signaling by overexpression of dominant-negative fragments of FAK reduces invasion of glioblastomas [11] and ovarian cancer cells [12]. FAK therefore represents an important target for the development of anti-neoplastic and anti-metastatic drugs.

Several kinase inhibitors are currently in clinical use for the treatment of cancer. Imatinib, an inhibitor of the Abl tyrosine kinase, was the first small molecule kinase inhibitor to be approved in the US (in 2001) and is now widely used for the treatment of chronic myeloid leukemia. Imatinib binds to the inactive conformation of the Abl kinase, which adopts a DFG flipped conformation (also termed DFG-out conformation) [13], [14]. The DFG flip is characterized by a rotation of the phi backbone torsion angle of the Asp in the DFG motif by approximately 180°. Much of the specificity of imatinib has been attributed to its recognition of the DFG flipped activation loop of Abl. Indeed, imatinib also efficiently inhibits the receptor tyrosine kinase c-Kit [15], [16], which also exhibits a DFG-out conformation in its autoinhibited state [17], whereas the much closer related Src family kinases are not efficiently targeted [16], [18]. Despite intense study, the selectivity of imatinib for Abl over Src is still not well understood. However, mutations in Src that were designed to destabilize the inactive Src conformation, and therefore potentially allow Src to adopt a DFG-out conformation with a lower energetic penalty, do exhibit increased affinity for imatinib [18].

Recently a novel bis-anilino pyrimidine compound, TAE226, was shown to efficiently inhibit growth and invasion of glioma and ovarian cancer cells [19]–[21] and to induce apoptosis in breast cancer cell lines [22]. Importantly, the compound efficiently increased survival rates of animals with glioma xenografts [20] or ovarian tumor cell implants [19]. TAE226 is a potent inhibitor of FAK (IC50 = 5.5 nM) and also inhibits insulin receptor (InsR) and insulin-like growth factor-I receptor (IGF-IR), albeit ∼10 fold less potently (IC50 = 44 nM for InsR and IC50 = 140 nM for IGF-IR) [20]. Since IGF-IR and its ligands IGF-I and IGF-II are frequently overexpressed in gliomas [23], [24], the dual specificity of TAE226 may increase its efficacy for the treatment of glioblastomas. TAE226 displays otherwise good selectivity against a panel of 30 kinases [20].

Here we report the crystal structures of the FAK kinase in complex with TAE226 and 3 related bis-anilino pyrimidine analogs. All compounds bind to the ATP binding pocket of the FAK kinase and the common core of the inhibitors interacts in an identical fashion with the kinase hinge region. The structures reveal that the carbonyl in the carbamoyl moiety of TAE226 and an analogous carbonyl in 2 of the 3 other compounds stabilize an unusual helical conformation of the DFG motif. This conformation is also found in the recently reported structure of FAK in complex with the inhibitor PF-562,271 [25], but differs substantially from DFG-out conformations seen in other kinases. Thus, this induced conformation is likely to confer selectivity against most kinases. Additionally, an analog of TAE226 that fails to induce the helical DFG conformation displays a 3-fold lower potency.

## Results

**Figure 1:**
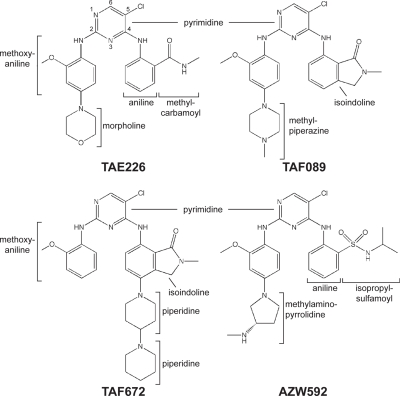
Schematic structures of the FAK specific inhibitors. The schematic structures of the FAK inhibitors used in this study are shown in a consistent orientation and a conformation that is derived from their conformation when bound to FAK.

**Figure 2:**
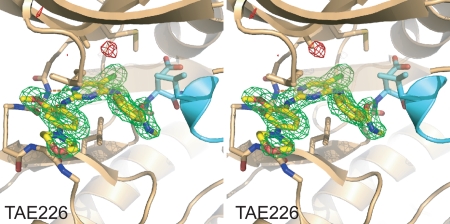
Difference electron density map for the FAK/TAE226 structure. Fo-Fc electron density calculated from experimental data and the final model with TAE226 removed is shown in stereo contoured at 3.5 σ. Positive electron density is colored green and negative density is colored red. FAK is shown as beige ribbon with its activation loop in cyan. TAE226 (yellow) and sidechains involved in inhibitor binding are shown in stick representation. The ribbons of residues 429–431 in the P-loop are rendered transparent for clarity. Difference electron density for TAE226 is well defined.

**Figure 3:**
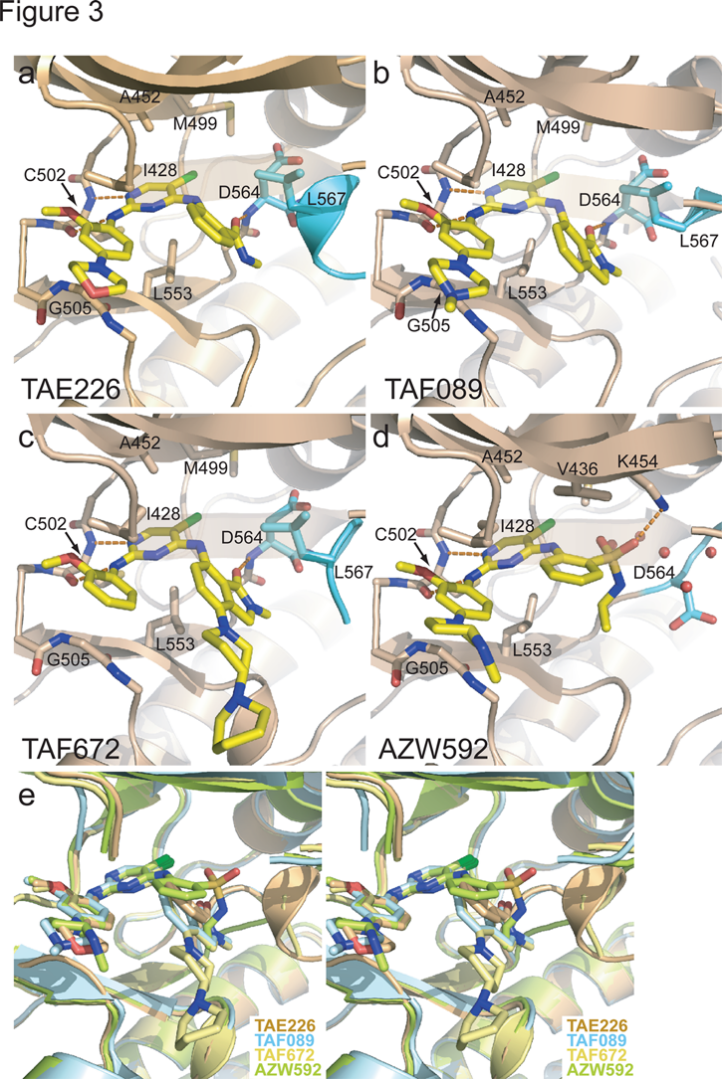
Drug binding mode of the FAK inhibitors. (a–d). The inhibitors TAE226 (a), TAF089 (b), TAF672 (c) and AZW592 (d) are shown bound to the active site of the FAK kinase (beige ribbon with activation loop in cyan). Key side chains and the inhibitors (yellow) are shown in stick representation. For clarity residues 429–431 in the P-loop are rendered transparent. (e) Stereoview of the superposition of the four structures displayed in (a–d). For clarity residues 429–431 in the P-loop are removed. The substitutions at the 4-aniline ring of TAE226, TAF089 and TAF672 induce a helical conformation of the DFG motif, whereas AZW592 bound FAK exhibits an extended activation loop in this region.

We solved co-crystal structures of the kinase domain of FAK in complex with TAE226 and three other bis-anilino-pyrimidine analogs (TAF089, TAF672 and AZW592) (Fig. 1, see Table 1 for crystallographic parameters). In all structures, the compound binds to the ATP binding pocket of the FAK kinase and electron density for the compounds is well defined (see Fig. 2 for difference electron density of TAE226). All compounds exhibit a common 5-chloro-2-ortho-methoxyanilino-4-anilinopyrimidine core structure, which binds in a nearly identical manner to the hinge region of the FAK kinase (Fig. 3). Two hydrogen bonds are formed between the backbone nitrogen and carbonyl group of Cys502 of the kinase hinge with nitrogens in the pyrimidine and 2-methoxyaniline moieties. The carbon atoms in the pyrimidine ring make hydrophobic contacts with Ala452 and Leu553, whereas carbons of the 2-methoxyaniline ring interact with Ile428 and Gly505 (Fig. 3a–d). The chlorine atom at the C5 position of the pyrimidine ring penetrates deepest into the ATP binding pocket and is located near the gatekeeper residue Met499. Bis-anilino pyrimidine compounds have previously been characterized as inhibitors of cyclin dependent kinases (CDK2, CDK4) [26]. Their mode of interaction with the kinase hinge region is highly similar to that observed here.

**Table 1:** Data collection and refinement statistics for FAK/inhibitor structures.

Compound	TAE226	AZW592	TAF089	TAF672
Data collection				
Space group	P2_1_	P2_1_	P2_1_	P2_1_
Cell dimensions				
*a*, *b*, *c* (Å)	44.1, 45.2, 67.5	44.1, 45.6, 66.4	45.7, 47.1, 63.0	44.5, 44.9, 66.4
α, β, γ (°)	90, 94.7, 90	90, 95.4, 90	90, 98.6, 90	90, 95.4, 90
Resolution (Å) *	50−2.0 (2.07−2.0)	50−2.3 (2.37−2.3)	50−1.65 (1.70−1.65)	50−2.6 (2.69−2.6)
R_merge_ *	10.7 (30.8)	11.0 (24.4)	9.6 (21.1)	18.0 (29.1)
I/σI*	11.2 (2.58)	10.8 (3.36)	13.2 (3.38)	8.0 (3.08)
Completeness (%)*	93.2 (61.0)	92.6 (70.1)	94.4 (59.7)	92.1 (63.4)
Redundancy*	3.9 (2.1)	3.4 (2.6)	4.2 (1.7)	4.2 (3.0)
Refinement				
Resolution (Å)	31.51−2.0	37.56−2.3	27.67−1.65	33.08−2.6
No. reflections	16 070	10 336	28 834	7222
R_work_/R_free_	17.9/23.6	20.8/27.2	18.8/21.7	21.5/26.2
No. atoms	2252	2194	2332	2128
Protein	2075	2084	2053	2064
Ligand	38	37	35	40
Water	139	73	244	24
Average B-factor	28.2	36.9	26.6	33.6
R.m.s deviations				
Bond lengths (Å)	0.013	0.010	0.008	0.013
Bond angles (°)	1.400	1.475	1.168	1.295

Each dataset was collected on one single crystal.

* Highest resolution shell is shown in parentheses.

**Figure 4:**
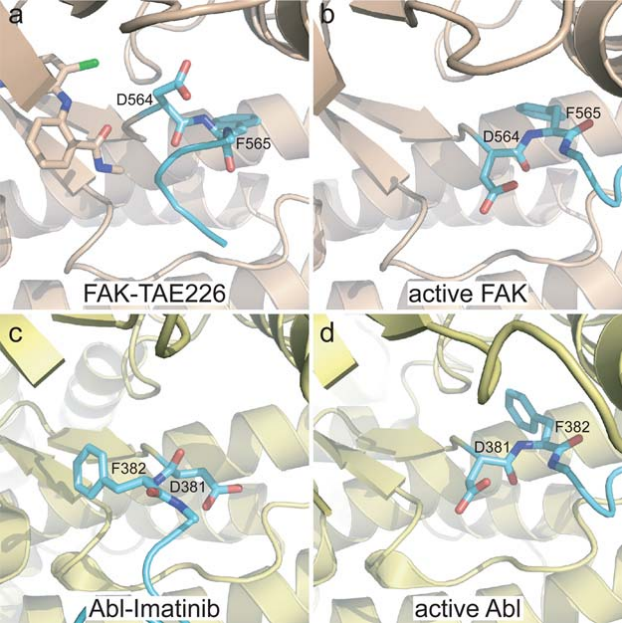
Partial DFG flip in TAE226 bound FAK. (a–b). The phi backbone torsion angle of D564 in TAE226 bound FAK (a) is rotated by approximately 110° compared to FAK in the active conformation (b) (pdb accession 2j0l). This results in the side chain of D564 pointing upwards towards the N-lobe. FAK is colored beige with its activation loop in cyan. TAE226 (a) and the DFG motif are shown in stick representation. For clarity, residues 429–434 in the P-loop and AMP-PNP in active FAK are removed. (c–d) In Abl, D381 of the DFG motif is flipped by approximately 180° in the imatinib bound form (c) (pdb accession 1opj) compared to the active conformation (d) (pdb accession 2g2i). Abl is colored pale yellow with its activation loop in cyan. The ligands imatinib (c) and ADP (d) as well as residues 251–255 in the P-loop are removed because they obstruct the view of the DFG motif.

The compounds are derivatized at both aniline rings. The morpholine (TAE226), methylpiperazine (TAF089), dipiperidine (TAF672) or methylaminopyrrolidine (AZW592) moieties in para position to the amine groups of the aniline rings make almost no protein contacts, consistent with their incorporation for improved pharmacokinetic properties of the respective compounds. The methyl carbamoyl moiety in ortho position to the amine in the 4-aniline ring of TAE226 is located near the DFG motif of the activation loop of the FAK kinase. Its carbonyl group forms a hydrogen bond with the backbone nitrogen of Asp564 of the DFG motif (Fig. 3a). The side chain of Leu567, the residue immediately following the DFG sequence, makes hydrophobic contacts with the 4-aniline ring. Interestingly, the interactions involving Asp564 and Leu567 stabilize a short helix that includes the DFG motif. This conformation of the DFG motif, which is induced by these contacts with TAE226, is similar to the conformation induced by the bis-amino pyrimidine FAK inhibiter PF-562,271 [25] and has to our knowledge only been observed in FAK. In order to adopt this helical turn the phi torsion angle of Asp564 is rotated by 113° compared to the active kinase domain (Fig. 4a, 4b). Together, the backbone rotations of Gly563 and Asp564 move the Cα of Asp564 by 3 Å closer to the N-lobe with its side chain closely approaching the N-lobe of the kinase.

The carbonyl within the isoindoline moieties of TAF089 and TAF672 superimpose well with the carbonyl in TAE226 and induce a similar helical turn at the DFG motif (Figs. 3a–c, 3e). In contrast, AZW592, which has a sulfamoyl group at the corresponding position, makes no significant interactions with the activation loop, but several water molecules fill the space between the compound and the activation loop (Fig. 3d). The activation loop in the AZW592 bound FAK kinase follows a non-helical path similar to the one observed in autoinhibited FAK [4]. This allows the gatekeeper residue Met499 to rotate its sidechain towards AZW592 and it makes van der Waals contact with the chlorine atom on the pyrimidine ring.

We evaluated the potencies of the FAK inhibitors by determining dose-response kinase inhibition curves and deriving IC50 values. As apparent from Table 2, TAE226 and TAF089 exhibit approximately 3-fold higher potencies than AZW592. Inspection of the crystal structures indicates that the increased potency may be attributed to the close contacts between the TAE226 and TAF089 compounds with the DFGL motif in the activation loop of FAK. IC50 values for TAF672 could not be reliably determined because of its low solubility in DMSO and aqueous buffers, however, it can be expected to mirror the potency of TAF089 since the moiety that interacts with the FAK kinase is identical for both inhibitors.

**Table 2:** IC50 values for FAK inhibitors.

Inhibitor	IC50 (nM)
TAE226	6.24±0.51
TAF089	7.70±1.36
AZW592	17.76±1.21

IC50 values were calculated by fitting the Hill slope model to inhibition curves determined at a concentration of 10 nM purified FAK kinase and 1 mM ATP.

## Discussion

Tyrosine kinases employ a remarkable variety of mechanisms in order to achieve autoinhibition (reviewed in [27]). The structural diversity of these inactive conformations can be an important determinant of drug specificity. In Abl, the inactive DFG-out conformation is exploited by the inhibitor imatinib to achieve high specificity. It is less obvious how specificity can be achieved for kinases like FAK that do not natively utilize unique conformations in proximity to the active site for regulation [4]. Interestingly, the FAK inhibitor TAE226 induces a DFG conformation in FAK that is distinct from that observed in Abl or other kinases. The phi torsion angle of Asp564 is rotated by approximately 110° compared to the active FAK kinase (Figs. 4a and 4b), whereas in other DFG flipped conformations, such as in the kinase domains of Abl [14], Kit [17], InsR [28], Flt3 [29], Csk [30], Raf [31] and p38 [32], the corresponding Asp is rotated by approximately 180° (Figs. 4c and 4d). This highlights that the conformational space accessible to kinases is not limited to conformations used during their native regulation cycle. The DFG/TAE226 contact in FAK is likely to confer high potency (Table 2) and selectivity [20].

The specificity profile of TAE226 [20] suggests that the semi-flipped conformation of the DFG motif is not accessible or not favored in most kinases. We note that the kinases with highest affinity for TAE226 (FAK, Pyk2, InsR, IGF-IR and ALK) all have a glycine preceding the DFG motif. Other than these kinases, of the 30 kinases tested by Liu et al. [20], only JAK2 and c-Raf have a Gly at this position. Presumably, other features of these kinases prevent potent inhibition by TAE226. Even though the Gly preceding the DFG motif is highly conserved in FAK, it is rare among all the tyrosine kinases in the human genome, occurring in only 17 out of 90 tyrosine kinases [33]. It is possible that TAE226 binding and the resulting Asp564 conformation are only energetically favored in kinases with GDFG motifs. Although in FAK the backbone torsion angles of Gly563 fall into the additional allowed region for non-glycine residues, it is possible that the freedom of a glycine residue is required to accommodate the conformational transition. Additionally, the side chain of any other residue is likely to clash against the methyl group of the methylcarbamoyl moiety of TAE226.

The bis-amino pyrimidine FAK inhibitor PF-562,271 was shown to induce a DFG conformation in FAK that is very similar to the TAE226 induced conformation [25]. Interestingly, the selectivity profile of PF-562,271 contrasts to the one of TAE226 in that PF-562,271 displays good selectivity against IGF-IR but inhibits several cyclin dependent kinases relatively efficiently. It is difficult to structurally explain these differences in specificity, but it suggests that by combining structural features of the two inhibitors the selectivity can be further improved.

In summary, our work highlights that in different kinases the DFG motif and its flanking sequence exhibit variable conformational plasticity. It is emerging that compounds that are able to lock this region into a conformation that is not favored by most other kinases can display good specificity profiles. Imatinib is an example of such a compound and it has successfully been translated into the clinic. TAE226 shows promising results in preclinical trials for the treatment of malignant gliomas [20], [21] and ovarian cancers [19]. Our work demonstrates that TAE226 locks the activation loop of FAK into a helical conformation, which to date has only been observed in FAK and appears to require a glycine residue immediately preceding the DFG motif. This sequence requirement contributes to the target selectivity of the compound, and thus to its efficacy and relatively limited toxicity [20].

## Materials and Methods

### Protein expression and crystallization

The kinase domain of avian FAK (FAK411-686) was expressed with a 6xhis-tag in insect Hifive™ cells (Invitrogen) using the baculovirus expression system and initially purified on Ni sepharose beads (GE Healthcare). Subsequently, the his-tag was removed with TEV protease and the FAK kinase was further purified by cation exchange (MonoS, GE Healthcare) and size exclusion (Superdex200) chromatography. The purified protein was concentrated to 8 mg/ml in 20 mM HEPES pH 7.0, 150 mM NaCl, 5% Glycerol and 2 mM TCEP. Inhibitor complexes were formed by incubating the FAK kinase with 1/50 volume of aqueous inhibitor suspensions at 100 mM for 3 h at 4°C. For crystallization, the complex was centrifuged in a microfuge and hanging drops were set up with an equal volume of 26% PEG4K, 200 mM LiSO_4_, 100 mM Tris pH 8.5, 10 mM TCEP. Crystals were transferred into a cryosolution containing 8% ethylene glycol, 32% PEG4000, 200 mM LiSO_4_, 100 mM Tris pH 8.5, and flash-frozen.

### Data collection and structure determination

Diffraction data were collected at beamline X29A at the National Synchrotron Light Source (Brookhaven National Laboratory) and processed with HKL2000 [34]. Phases were calculated using the molecular replacement program PHASER [35] using the kinase domain (pdb accession 1MP8) [36] as molecular search probe. Refinement was performed using the program Refmac [37] and manual rebuilding was carried out with Coot [38]. Final R-factors were 17.9/23.6 (R_work_/R_free_) for FAK/TAE226. For a complete summary of data collection and refinement parameters see Table 1.

### Inhibition curves and IC50 value

An anti-phosphotyrosine based ELISA assay was used to determine kinase inhibition curves. Briefly, kinase reactions were performed with 10 nM FAK kinase (FAK411-686), 1 mM ATP, 5 mM MgCl_2_ and 1 µM FAK31-405 as substrate (the FAK31-405 fragment contains the Tyr397 autophosphorylation site). Inhibitor concentrations between 0.05 and 100 nM were added and reactions were incubated for 30 min at RT. Reactions were stopped with 100 mM EDTA and the reaction mixtures were transferred to 96 well Maxisorp plates (Fisher Scientific) for immobilization. Following blocking with 5% BSA, plates were incubated with 1∶2000 PY20 antibody conjugated with HRP. Plates were washed, developed with TMB substrate (Calbiochem) and the absorption was monitored at 650 nm for 5 min using a Spectra MAX Plus spectrometer (Molecular Devices). Kinase activity was determined for each inhibitor concentration 6 times and mean values were plotted against inhibitor concentration. IC50 values were determined by fitting the Hill slope model to the inhibition curves.
